# Fatal Algaemia in Patient with Chronic Lymphocytic Leukemia

**DOI:** 10.3201/eid1507.090373

**Published:** 2009-07

**Authors:** Philippe Lanotte, Gaelle Baty, Delphine Senecal, Caroline Dartigeas, Eric Bailly, Thanh Hai Duong, Jacques Chandenier, Alain Goudeau

**Affiliations:** Centre Hospitalier Régional Universitaire de Tours, Tours, France (P. Lanotte, G. Baty, D. Senecal, C. Dartigeas, E. Bailly, T.H. Duong, J. Chandenier, A. Goudeau); Université François Rabelais, Tours (P. Lanotte, G. Baty, T.H. Duong, J. Chandenier, A. Goudeau)

**Keywords:** Prototheca, immunocompromised patient, parasites, leukemia, algae, letter

**To the Editor:** *Prototheca* species are achlorophyllic lower algae, ubiquitous in nature, which can cause human infections, particularly in immunocompromised patients (*1*). Human protothecosis is mostly caused by *P. wickerhamii* and *P. zopfii*. Although such infections are infrequent, they can manifest themselves clinically as cutaneous lesions, olecranon bursitis, and, even more rarely, as disseminated or systemic infections (*1*). These infections occur in severely immunocompromised patients, such as persons with AIDS, or patients undergoing extensive treatment, such as cancer treatment or organ transplantation (*1*–*4*). We describe a fatal case of *P. wickerhamii* algaemia in a patient with chronic lymphocytic leukemia.

In July 2007, a 79-year-old man, who had been monitored since 1993 for stage C chronic lymphocytic leukemia (*5*), was hospitalized July 13–20 for a depressive syndrome with fever, asthenia, and weight loss (3 kg over 2 months). Blood and urinary cultures on admission were sterile. The patient was hospitalized again on July 30 for fever (39°C), anorexia, and diarrhea, with ≈7 stools per day. He had lost 10 kg in 2 weeks. Blood cultures for bacteria (in BD Bactec Plus Aerobic/F and BD Bactec Lytic Anaerobic/F vials; Becton Dickinson, Le Pont de Claix, France) and fungi (BD Bactec Mycosis IC/F; Becton Dickinson) and stool cultures for bacteria were negative. Blood cultures were incubated in a Bactec 9240 instrument (Becton Dickinson). *Aspergillus fumigatus* was found in a bronchoalveolar lavage specimen, but no *Aspergillus* galactomannan antigen was detected in blood.

The patient was treated with piperacillin-tazobactam, ciprofloxacin, acyclovir, voriconazole, and loperamide. Voriconazole (400 mg/day) was used from day 17 to day 27. On day 21, *Cryptosporidium parvum* was detected on parasitologic stool examination. Symptoms persisted on day 26, with strong asthenia and deterioration of general state. At that time, the leukocyte count was 178 × 10^9^/L with 3.56 × 10^9^/L polymorphonuclear neutrophils and 172 × 10^9^/L lymphocytes. Three peripheral blood samples were cultured for detection of bacteria and fungi. On day 27, septic shock developed in the patient. A blood culture showed an *Escherichia coli* strain susceptible to piperacillin-tazobactam, aminoglycosides, and quinolones. Amikacin was added to the treatment regimen. Nonetheless, the patient died on day 28.

Two blood cultures for bacteria in aerobic vials grew the day of the patient’s death, but tests of blood cultures for fungus remained negative. After Gram staining, gram-positive spherical unicellular organisms were observed (Figure). After 48 hours of incubation, creamy, yeast-like colonies grew on chocolate agar (bioMérieux, Marcy l’Etoile, France), but not on Sabouraud agar containing gentamicin and chloramphenicol (Becton Dickinson). Microscopy and the API 20C AUX system (bioMérieux) identified *P. wickerhamii.*

**Figure:**
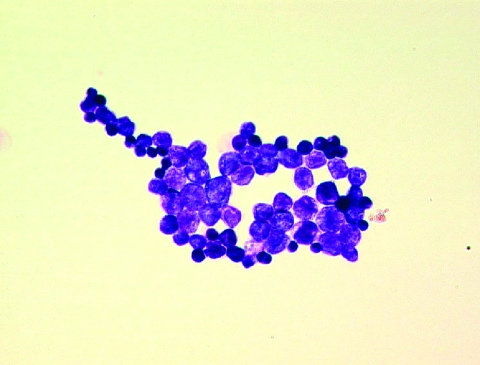
Gram-positive spherical unicellular organisms in a blood culture from a 79-year-old man with chronic lymphocytic leukemia. Magnification ×1,000.

Sequencing the 18S rDNA with the primers Pw18SF 5′-TCAAAAAGTCCCGGCTAATCTCGTGC-3′ and Pw18SR 5′-CGCTTTCGTGCCTCAATGTCAGTGTT-3′ confirmed the identification. The sequence of the amplified product was compared with sequences published in the database of the National Center for Biotechnology Information (Bethesda, MD, USA). The most likely identification, according to BLAST analysis (www.ncbi.nlm.nih.gov/blast/Blast.cgi), was *P. wickerhamii*.

In vitro susceptibility tests were performed by the Etest method (AB Biodisk, Solna, Sweden), on RPMI agar. *P. wickerhamii* was found to be susceptible to amphotericin B and posaconazole, with MICs of 0.047 μg/mL and 0.012 μg/mL, respectively. By contrast, it was resistant to fluconazole (MIC>256 μg/mL), voriconazole (MIC>32 μg/mL), and caspofungin (MIC>32 μg/mL). It was also susceptible to gentamicin (MIC = 0.25 μg/mL) but resistant to amikacin (MIC>24 μg/mL).

However, the patient died before the algae were detected in the blood culture vials. In this case, antifungal treatment based on voriconazole use was empiric and ineffective. Some authors have described a successful treatment on localized protothecosis with voriconazole (*6*). Amphotericin B currently seems to be most effective agent, although the best treatment remains a matter of debate (*1*,*4*,*7*). Although in vitro susceptibility test results are not necessarily well correlated with results obtained in vivo, the low MIC of posaconazole reported here may be of interest in clinical practice (*8*). In the laboratory, use of selective yeast media, such as Sabouraud plus gentamicin, or Mycosis IC/F vials for blood culture, which contain chloramphenicol and tobramycin, may make it difficult to detect *Prototheca* spp.*,* which are susceptible to these antimicrobial drugs.-

In patients with algaemia, *Prototheca* spp. are often associated with bacteria, viruses, or yeasts which cause co-infections (*1*), as in this case, in which the alga was associated with *E. coli*. This association is probably the result of disseminated protothecosis in severely immunocompromised patients, and the alga may cross digestive or cutaneous barriers. Reasons for septic shock or death are unclear for most associations of pathogens (*2*,*4*). *Prototheca* spp. are found in various reservoirs, including the environment, animals, and food (*1*). In the case described here, the infection may have originated from a contaminated well used to obtain water for the patient’s kitchen garden. However, we were unable to test this hypothesis.

Disseminated protothecosis is currently rare but, due to the algae’s ubiquitous nature, increasing use of immunosuppressive therapy, and increasing incidence of hematologic malignancy, *Prototheca* spp. may emerge as opportunistic pathogens. *Prototheca* spp. should also be considered as an emerging cause of systemic infection in immunocompromised patients.
